# An Immunoassay for Dibutyl Phthalate Based on Direct Hapten Linkage to the Polystyrene Surface of Microtiter Plates

**DOI:** 10.1371/journal.pone.0029196

**Published:** 2011-12-27

**Authors:** Chenxi Wei, Shumao Ding, Huihui You, Yaran Zhang, Yao Wang, Xu Yang, Junlin Yuan

**Affiliations:** 1 Hubei Key Laboratory of Genetic Regulation and Integrative Biology, College of Life Sciences, Huazhong Normal University, Wuhan, China; 2 College of Life Sciences, Fudan University, Shanghai, China; University of Kansas Medical Center, United States of America

## Abstract

**Background:**

Dibutyl phthalate (DBP) is predominantly used as a plasticizer inplastics to make them flexible. Extensive use of phthalates in both industrial processes and other consumer products has resulted in the ubiquitous presence of phthalates in the environment. In order to better determine the level of pollution in the environment and evaluate the potential adverse effects of exposure to DBP, immunoassay for DBP was developed.

**Methodology/Principal Findings:**

A monoclonal antibody specific to DBP was produced from a stable hybridoma cell line generated by lymphocyte hybridoma technique. An indirect competitive enzyme-linked immunosorbent assay (icELISA) employing direct coating of hapten on polystyrene microtiter plates was established for the detection of DBP. Polystyrene surface was first oxidized by permanganate in dilute sulfuric acid to generate carboxyl groups. Then dibutyl 4-aminophthalate, which is an analogue of DBP, was covalently linked to the carboxyl groups of polystyrene surface with 1-ethyl-3-(3-dimethylaminopropyl) carbodiimide hydrochloride (EDC). Compared with conjugate coated format (IC_50_ = 106 ng/mL), the direct hapten coated format (IC_50_ = 14.6 ng/mL) improved assay sensitivity after careful optimization of assay conditions. The average recovery of DBP from spiked water sample was 104.4% and the average coefficient of variation was 9.95%. Good agreement of the results obtained by the hapten coated icELISA and gas chromatography-mass spectrometry further confirmed the reliability and accuracy of the icELISA for the detection of DBP in certain plastic and cosmetic samples.

**Conclusions/Significance:**

The stable and efficient hybridoma cell line obtained is an unlimited source of sensitive and specific antibody to DBP. The hapten coated format is proposed as generally applicable because the carboxyl groups on modified microtiter plate surface enables stable immobilization of aminated or hydroxylated hapten with EDC. The developed hapten coated icELISA can be used as a convenient quantitative tool for the sensitive and accurate monitoring DBP in water, plastic and cosmetic samples.

## Introduction

**Figure 1:**
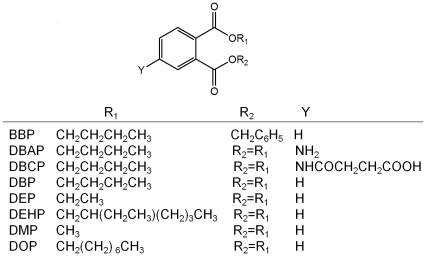
Structures of DBP and analogues.

Dibutyl phthalate, commonly referred to as DBP (Fig. 1), is predominantly used as a plasticizer in nitrocellulose lacquers and polyvinyl chloride (PVC) plastics to make them flexible. It is also used as a solvent for dyes and pesticides. In addition, DBP is one kind of industrial raw materials for anti-foaming agent, latex adhesives and textile fiber lubricants. These materials are used to make many products that we use every day such as plastics, paints, glue, insect repellents, perfume, hair spray, nail polish and so on.. Release of DBP to the envitonment can occur during its production and the incorporation of the phthalate into plastics, adhesives, or dyes. Because DBP is not bound to the final product, it can move out of products into the environment over long periods of time. Therefore, DBP is widespread in the environment. Humans can be exposed to DBP through air, water, food, or skin contact with plastics which contain DBP [1]. In recent years, DBP is considered to be an environmental endocrine disruptor, with reproductive toxicity, developmental toxicity and potential carcinogenic effects [2].

In order to better determine the level of pollution in the environment and evaluate the potential adverse effects of exposure to DBP, methods for DBP determination must be developed. Several methods have been reported for the determination of DBP using a variety of techniques, including gas chromatography coupled with mass spectroscopy (GC-MS) and high performance liquid chromatography (HPLC) [3]–[5]. Although the chromatographic techniques provide a low level of detection for phthalates, they are time consuming and have high instrumentation costs. On the contrary, immunoassay is a fast, simple, and economic analytical method. Because of its strong selectivity and sensitivity, efforts for sample cleanup can be reduced to a minimum, which makes the immunoassays highly convenient tools for high throughput studies for a large number of samples in a short period of time [6].

Zhang et al has reported a competitive fluorescence immunoassay for determination of DBP based on polyclonal antibody [7]. From the competitive inhibition standard curve for the detection of DBP they established, 1000 µg/L (the maximum concentration they used) of DBP even did not cause 50% of the maximal fluorescence quenching although they stated that the limit of detection (LOD) was 0.02 µg/L. Yanaihara et al developed a direct competitive enzyme-linked immunosorbent assay (ELISA) for phthalates also based on polyclonal antibody [8]. Nevertheless, polyclonal antibody is restricted by immunized animals and cannot be produced unlimitedly. Furthermore, the character of polyclonal antibody from different immunized animals is different, which made it difficult to standardize the measurement.

In most hapten based ELISAs, haptens are usually bound to polystyrene microtiter plates indirectly by coating the wells with hapten–protein conjugates, since direct attachment of haptens to a polystyrene surface is not possible due to the lack of available functional groups on polystyrene. However, the adsorption of this conjugates to a polystyrene surface inevitably involves significant conformational changes to create large-scale hydrophobic contacts. Conformational changes would influence hapten presentation for the reason that low molecular weight compounds with small size could be easily screened by protein macromolecule. Moreover, the formation of the hapten-protein conjugates is not always reproducible, which is difficult to make assay standardization. To avoid these drawbacks, methods for direct attachment of some hapten on polystyrene have been reported [9]–[11].

In this study, polystyrene was oxidized to generate reactive carboxyl groups, then directly linked dibutyl 4-aminophthalate (DBAP), an aminated hapten with structural feature of DBP, to modified polystyrene surface with1-ethyl-3-(3-dimethylaminopropyl) carbodiimide hydrochloride (EDC). Highly sensitive and specific monoclonal antibody (MAb) against DBP were produced and used in the present hapten coated ELISA format showing high degree of assay sensitivity for DBP.

## Materials and Methods

### Materials

Butyl benzyl phthalate (BBP), DBP, diethyl phthalate (DEP), di(2-ethylhexyl) phthalate (DEHP), dimethyl phthalate (DMP), di(n-octyl) phthalate (DOP) (Fig. 1), bovine serum albumin (BSA), ovalbumin (OVA), EDC, 2-(N-morpholino) ethanesulfonic acid (MES), complete and incomplete Freund's adjuvant, hypoxanthine-aminopterin-thymidine (HAT) and hypoxanthine-thymidine (HT) media supplement (50×), polyethylene glycol solution and mouse monoclonal antibody (MAb) isotyping reagents were purchased from Sigma-Aldrich Co. (St. Louis, MO, USA). Horseradish peroxidase conjugated goat anti-mouse IgG (HRP-anti-mIgG) was from Jackson ImmunoResearch Laboratories, Inc. (West Grove, USA). Dulbeco Modified Eagle's Medium (DMEM), fetal bovine serum and penicillin–streptomycin were obtained from Invitrogen Co. (California, USA). All other chemicals and solvents were of analytical grade or better were purchased from Sinopharm Chemical Reagent Co. (Shanghai, China). Specific Pathogen Free Female BALB/c mice (8∼10 weeks old) were obtained from the Experimental Animal Center of Hubei (Wuhan, China), and housed under standard laboratory conditions (temperature, 24±2°C; humidity, 50±10% and 12-h day/night cycles). The mice were given free access to a standard chow diet and drinking water ad libitum. Our animal work have been conducted according to relevant national and international guidelines and the study protocol was approved by the Office of Scientific Research Management of Huazhong Normal University, with a Ratification on Application for the Use of Animals (approval ID: CCNU-SKY-2010-003) dated March 3, 2010. The murine myeloma cell line SP2/0 was obtained from Preservation Center of Wuhan University (Wuhan, China). An S-3100 UV-vis spectrophotometer (Scinco, Korea) was used to collect UV data. ELISA was carried out in 96-well polystyrene microtiter plates (Canada JET Biochemicals Int. Guangzhou, China). Immunoassay absorbance was read by a PowerWave XS Microplate Spectrophotometer (Bio-Tek Instruments, Inc. Vermont, USA).

### Synthesis of derivatives of DBP

Aminated hapten DBAP and carboxylated hapten DBCP were prepared according to Yanaihara et al [8]. DBAP was synthesized via esterification of 4-nitrophthalic acid with butanol followed by reduction with purified zinc dust and concentrated hydrochloric acid. DBCP was prepared by the reaction of DBAP with succinic anhydride. The structures were confirmed by ^1^H NMR (400 MHz, CDCl_3_). DBAP, δ(ppm): 7.72 (1H, d, ArH), 7.26 (1H, s, ArH), 6.74 (1H, dd, ArH), 4.29 (2H, t, OCH_2_CH_2_CH_2_CH_3_), 4.23 (2H, t, OCH_2_CH_2_CH_2_CH_3_), 4.11 (2H, brs, NH_2_), 1.73∼1.63 (4H, m, 2OCH_2_ CH_2_CH_2_CH_3_), 1.46∼1.39 (4H, m, 2 OCH_2_CH_2_ CH_2_CH_3_), 0.96 (6H, t, 2CH_3_). DBCP, δ(ppm): 11.5 (1H, brs, COOH), 8.44 (1H, s, ArH), 8.12 (1H, brs, NH), 8.01 (1H, d, ArH), 7.92 (1H, d, ArH), 4.29 (2H, t, OCH_2_CH_2_CH_2_CH_3_), 4.23 (2H, t, OCH_2_CH_2_CH_2_CH_3_), 4.11 (2H, brs, NH_2_), 2.64 (2H, t, CH_2_CH_2_COOH), 2.47 (2H, t, CH_2_ CH_2_COOH), 1.73∼1.63 (4H, m, 2OCH_2_ CH_2_CH_2_CH_3_), 1.46∼1.39 (4H, m, 2 OCH_2_CH_2_ CH_2_CH_3_), 0.96 (6H, t, 2CH_3_).

### Preparation of hapten-protein conjugates

DBAP was linked to BSA or OVA through diazotization to generate artificial antigen DBAP-BSA or DBAP-OVA according to the method described by Ahn et al [12] with some modifications. DBAP (0.2 mmol) dissolved in 0.2 mL of dioxane was added to a stirred solution containing 2 mL of 1 mol L^−1^ HCl and 1.8 mL of dioxane. The mixture was stirred in an ice bath as 0.2 mL of 1 mol L^−1^ sodium nitrite was added dropwise and reacted for 30 min. Then the diazo salt solution was added dropwise to 10 mL of 0.2 mol L^−1^ borate buffer (pH 9.0) which dissolved 60 mg of BSA or OVA. The mixture was stirred at 4°C for 3 h. The orange conjugate solution was centrifuged at 10 000 g for 5 min and the supernatant passed through a Sephadex G-25 column to remove unconjugated DBAP. DBCP was conjugated to BSA or OVA to generate artificial antigen DBCP-BSA or DBCP-OVA by the active ester method according to the method of Lei et al [13]. All the hapten-protein conjugates were characterized by UV-vis spectrometry.

### Immunization of BALB/c mice

BALB/c mice were immunized with the hapten-protein conjugates (Mice 1, 2 and 3 for DBAP-BSA, Mice 4, 5 and 6 for DBAP-OVA, Mice 7, 8 and 9 for DBCP-BSA, and Mouse 10, 11 and 12 for DBCP-OVA). At the first day, each immunogen (100 µg) in 100 µL of PBS (10 mmol/L phosphate buffer, pH 7.4, with 137 mmol/L NaCl and 2.7 mmol/L KCl) was emulsified with an equal volume of complete Freund's adjuvant, and the emulsion was then injected subcutaneously at multiple sites on the neck and back of mice. On the 3rd day, repeat the above injection once to strengthen the first immunization stimulation. On the 28th and 49th day, the mice were boosted with an additional immunogen (50 µg) in 100 µL of PBS emulsified an equal volume of incomplete Freund's adjuvant [14]. Mice were tail-bled 7–10 days after the third and the fourth injection. Indirect noncompetitive ELISA (inELISA) and icELISA with 1.0 µg/mL heterologous coating protein conjugates were used to determine the titer and sensitivity of antibody in the serum. The mouse that generated polyclonal antibody with a combination of the best sensitivity and the highest titer received a final intraperitoneal injection of 200 µg of immunogen in 200 µL of PBS, 3 days before cell fusion.

### Cell Fusion, hybridoma culture and selection

SP 2/0 murine myeloma cells were cultured in DMEM supplemented with 15% fetal bovine serum and 1% penicillin–streptomycin. Cell fusion and hybridoma culture procedures were carried out essentially as described by James [15]. Mice splenocytes were mixed with SP 2/0 cells at the ratio of 5∶1 and centrifuged at 450 g for 5 min. One milliliters of polyethylene glycol solution at 37°C was dropped to the cell pellet over 1 min. The cell pellet was rotated for further 1 min, then 1 mL of DMEM added over 1 min, 2 mL of DMEM over 2 min. Finally, 12 mL was added over 2 min. After centrifugation at 450 *g* for 5 min, the cells were pelleted again, resuspended in HAT medium (DMEM containing 15% fetal bovine serum, 1% penicillin–streptomycin and 2% HAT media supplement) and plated into 96-well plates in which normal mouse peritoneal exudate cells (as feeder cells) had already been cultured 1 day before fusion. The colonies were fed every fifth day with freshly prepared HAT medium. Two weeks later, the hybrids were cultured in HT medium (DMEM containing 15% fetal bovine serum, 1% penicillin–streptomycin and 2% HT media supplement) for a few days prior to using normal medium. When the colonies reached at least fifth-confluence in the well, hybridomas were screened for specific MAb against DBP by simultaneous inELISA and icELISA described later. The ratio of both absorbances was used as the criterion for selecting the best antibody-secreting clones. The selected hybridomas were cloned by limit dilution. Mouse MAb isotyping reagents were used to determine the isotype of MAb according to the manufacturer's protocol. Giemsa staining was performed to analyze the hybridoma chromosomes [16].

### Large-scale production of monoclonal antibody in ascites fluid

Female BALB/c mice, 10 weeks old, were injected intraperitoneally with 0.5 mL of paraffin oil 7–10 days before receiving an intraperitoneal injection of 2×10^6^ hybridoma cells suspended in PBS. Ascites fluid developed 2–3 weeks after the injection of the cells and was collected every other day for 6 days [15]. The ascites fluid was centrifuged at 450 g for 5 min to remove cell pellet. The supernatant was diluted with equal volume of PBS and appropriate amount of silica dioxide (15 mg/mL) was added to the supernatant and incubated at room temperature for 30 min with rotation. The IgG from the pretreated ascites fluid was purified by caprylic acid/ammonium sulfate precipitation twice and dialysis and then stored at −70°C [17], [18]. The concentration and purity of purified MAb were determined by taking absorbance at 280 nm and SDS-polyacrylamide gel electrophoresis, respectively.

### Conjugate coated indirect noncompetitive ELISA

Checkerboard titration, in which purified MAb was titrated against varying amounts of the coating hapten-conjugate was used to measure the reactivity of antibodies and to select an appropriate coating concentrations and antibody dilutions for icELISA [19]. The checkerboard assays were performed with inELISA. Microtiter plates were coated with 100 µL/well of hapten-protein conjugates (0, 0.125, 0.25, 0.5, and 1 µg/mL) in 0.05 mol/L carbonate buffer (pH 9.6) and incubated overnight at 4°C. The plates were washed three times with PBST (0.01 mol/L PBS containing 0.05% Tween-20, pH 7.4, 200 µL/well) and were blocked with 3% (w/v) skim milk powder (SMP) in PBS (250 µL/well) for 1 h at 37°C. After another washing step, 100 µL/well of purified anti-DBP MAb (1∶1000, 1∶2000, 1∶4000, 1∶8000, 1∶16000, and 1∶32000) diluted in PBS containing 0.1% BSA was added. After incubation for 1.5 h at 25°C, the plates were washed. Subsequently, the plates were incubated for 1 h at 37°C with 10000-fold diluted HRP-anti-mIgG in PBS containing 0.1% BSA (100 µL/well). After another washing step, 100 µL/well of freshly prepared O-phenylenediamine (OPD) solution (0.4 mg/mL OPD and 0.012% H_2_O_2_ in 0.05 mol/L citrate-phosphate, pH 5.0) was added. The reaction was stopped with 2 mol/L sulfuric acid (50 µL/well) after an incubation of 15 min at room temperature keeping away from light. The absorbance was recorded at a primary wavelength of 492 nm with a reference wavelength of 630 nm.

### Conjugate coated indirect competitive ELISA

An icELISA was used to assess the sensitivity of the MAb to free DBP and the cross-reactivities of structurally related compounds to the antibody. For competition, 50 µL of standards or diluted samples in assay buffer were placed in the wells, and 50 µL of the anti-DBP MAb diluted with assay buffer was added. After incubated for 1.5 h at 25°C, the plate was washed. The following procedure was followed as described for the inELISA. With the icELISA format, analytes that do not react with the antibodies would produce the maximum absorbances; conversely, analytes that do react with the antibody would decrease in percentage of absorbance. Standards and samples were run in triplicate wells, and the mean absorbance values were processed. Standard curves were obtained by plotting absorbance against the logarithm of analyte concentration and fitted with a logistic four-parameter equation *y* = (*A*−*D*)/[1+(*x*/*C*)*^B^*]+*D*, where *A* is the absorbance value with no analyte present, *B* is the slope near the midpoint inhibition concentration, *C* is the concentration of analyte giving 50% inhibition (IC_50_), and *D* is the minimum absorbance value at infinite concentration of the analyte [20], [21]. Dynamic range was established between the concentrations producing 20% and 80% inhibition (IC_20_–IC_80_ or 80%–20% of the maximum response). Limit of detection (LOD) was determined as the concentration corresponding to 10% inhibitory concentration (IC_10_ or 90% of the maximum response) [22]–[24].

### Optimization of icELISA

The influence of several experimental parameters on assay characteristics was examined in order to improve the sensitivity of the immunoassay. Concentrations of coating antigen and dilutions of MAb were determined by checkerboard titration. Other different experimental parameters such as organic solvents, pH, ionic strength, and detergent in assay buffer, and blocking agents were studied sequentially in this order using the above described icELISA protocol. Each time an evaluated parameter was changed, the new value was used for the evaluation of the next condition. Criteria used to evaluate the assay performances were the absorbance of zero concentration of DBP (A_max_) and IC_50_.

### Hapten coated ELISA

**Figure 2:**
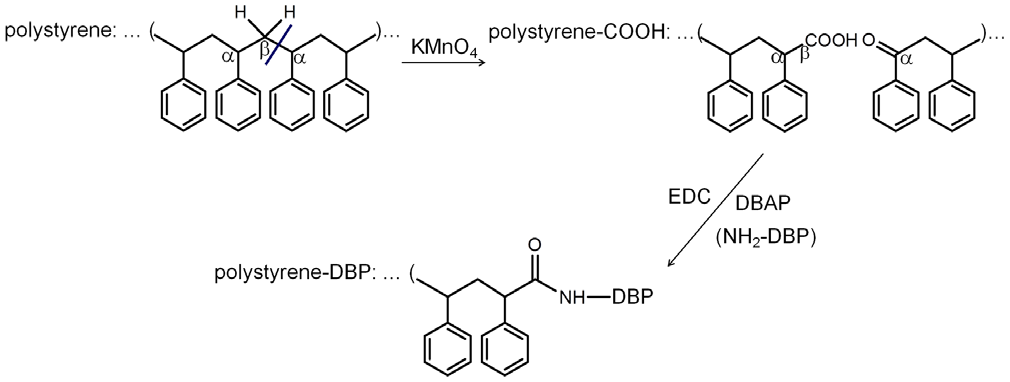
Scheme of one possible reaction in permanganate oxidation of polystyrene and its subsequent reaction with DBAP.

Polystyrene surface was first carboxylated by permanganate oxidation. Microwells were filled with a solution of 5% KMnO_4_ in 0.6 mol/L H_2_SO_4_ (150 µL/well) for 60 min at 60°C. The plates were washed with 6 mol/L HCl (3×5 min, 200 µL/well) to remove the brown manganese oxide and rinsed three times with distilled water giving polystyrene-COOH microwells [25]. Then 150 µL of EDC and hapten DBAP in 0.1 mol/L MES buffer (pH 5.0) were placed in the wells and incubated overnight under shaking (Fig. 2). The subsequent procedures were the same as conjugate coated inELISA or icELISA under optimal condition. The essential steps in direct attachment of hapten DBAP to polystyrene surface such as concentrations of EDC and conjugation temperatures were determined by the amount of antibody on the modified polystyrene surface measured by inELISA with coating concentration of 2 µg/mL DBAP and MAb dilution of 1∶2000. The optimal coating concentration of DBAP and dilution of MAb were determined by checkerboard titration.

### Cross-reactivity Determination

To determine the specificity of MAb produced, the ability of the MAb to recognize several structurally related compounds (analogues) was tested by performing hapten coated icELISA and determining their respective IC_50_ values. The cross-reactivity (CR) values were calculated according to the following equation CR (%) = IC_50 DBP_/IC_50 analogue_×100 [23], [26].

### Analysis of DBP in Spiked Water Samples

For recovery studies, water samples from a tap in the laboratory and East Lake in Wuhan were collected. DBP was spiked into these samples in levels of 0, 10, 50, and 100 ng/mL. Spiked samples were mixed with an equal volume of MAb solution prepared in a two-fold concentrated assay buffer and hapten coated icELISA was used to determine DBP concentrations in the spiked water samples [27].

### Detection of DBP in some consumer products

Each of the food storage bags and disposable plastic drinking cups was cut into pieces (0.5 cm^2^) and then weighed 0.5 g. A certain brand of nail polish was also weighed 0.5 g. After that 5.0 mL of acetonitrile was added to the pieces or nail polish and ultrasonically vibrated for 30 min [4]. Another extraction procedure in stoppered glass tube was carried out at room temperature overnight. The extract (2 mL) was evaporated using a centrifugal evaporator to remove acetonitrile and then dissolved in 2 mL of assay buffer [8]. The resulting extract was used as a test sample. The amount of DBP in the test sample was measured according to the method of the described hapten coated icELISA. The accuracy of the assay was confirmed by GC-MS method according to Zhang et al [4].

## Results

### Characterization of Artificial antigen

**Figure 3:**
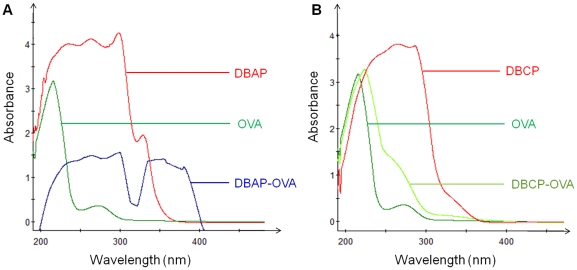
UV absorbance spectra of DBAP, OVA, DBAP-OVA (A) and DBCP, OVA, DBCP-OVA (B). All the concentrations of the tested substances were 0.5 mg/mL.

The aminated hapten DBAP and carboxylated hapten DBCP were synthesized and conjugated to the carrier proteins in order to produce suitable immunogens and coating antigens. UV-vis spectra of DBAP, DBCP, OVA, DBAP-OVA and DBCP-OVA are shown in Fig. 3. DBAP possessed a strong absorbance in the range of 210–305 nm and absorbance peaks at 234.5 nm, 262.8 nm, 297.7 nm and 328.9 nm. DBCP had a strong absorbance in 210–305 nm. OVA had obvious absorbance peaks at 215.3 nm and 271.7 nm. The characteristic absorbance of DBAP (210–305 nm) and unique absorbance in 330–385 nm were observed on the UV-vis spectrum of DBAP-OVA. The absorbance of DBCP-OVA showed a red-shift maximum at 223 nm compared with the 215.3 nm maximum for OVA and a downward convex curve in 245–300 nm. Both artificial antigens exhibited characteristic absorbance of the corresponding hapten or carrier protein and unique absorbance of their own, which indicated the successful conjugation between the haptens and OVA. The conjugates DBAP-BSA and DBCP-BSA gave UV spectra similar to that of DBAP-OVA and DBCP-OVA, respectively.

### Screening of polyclonal antisera

Two DBP derivatives DBAP and DBCP (Fig. 1), which differed in the spacer length, were used for conjugation with carrier proteins. The results of the titers and sensitivities of antisera are shown in Table 1. Antibodies raised against DBCP-protein conjugates showed higher titer but much lower sensitivity than DBAP-protein conjugates. DBAP-OVA promoted antibodies with higher sensitivity than DBAP-BSA. Mouse 2 exhibited the best combination of polyclonal antibody with high titer and low IC_50_ was selected for as spleen donors for the hybridoma production.

**Table 1:** Titers and sensitivity of antisera of immunized mice.

Immunogen/Coating antigen^a^	Mouse	Titer^b^	IC_50_ (ng/mL)^c^
DBAP-OVA / DBAP-BSA	1	4000	395
	2	8000	432
	3	16000	513
DBAP-BSA / DBAP-OVA	4	16000	1624
	5	8000	1172
	6	8000	1335
DBCP-OVA / DBCP-BSA	7	16000	>10^7^
	8	32000	>10^7^
	9	16000	>10^7^
DBCP-BSA / DBCP-OVA	10	16000	>10^7^
	11	16000	>10^7^
	12	8000	>10^7^

*^a^*The coating concentration was 1 µg/mL.

*^b^*The last dilution of serum that gave an absorbance at 492 nm of >1.0 was judged as the titer.

*^c^*IC_50_ represents the mean of 3 wells replicates.

### Production and characterization of hybridomas secreting MAb to DBP

The spleen cells from Mouse 2 were fused with Sp2/0 myeloma cells to produce hybridoma cells. Results of the hybridoma selection are summarized in Table 2. The inELISA and icELISA with 1 µg/mL DBAP-BSA as a coated antigen was used for screening the hybridoma cells, which were able to produce MAb specific to DBP. Of the 352 wells examined, only three clones gave IC_50_<5 µg/mL DBP; among them, the clone 4E9 showed the highest affinity for DBP. After five cloning, a stable hybridoma cell line (from the clone 4E9) secreting anti-DBP MAb with high titer and sensitivity was obtained and was selected for the production of ascites fluid. The isotype of MAb produced by hybridoma 4E9 was found to be IgG1. Chromosome counts revealed that the chromosome number of the hybridoma 4E9 was 96–105 with an average of 100, which almost was the sum of the chromosome number of mouse spleen cell (40) and Sp2/0 (60–68).

**Table 2:** Summary of the results of cell fusion and hybridoma selection.

Fusion parameters	No. of wells	Ratio (%)
seeded	352	-
fused	321	91.2
positive^a^	222	69.2
competitive^b^	3	0.935
no. of cloned hybridomas^c^	1	-

*^a^*Wells with antibodies that recognized 1 µg/mL of DBAP-BSA conjugate by inELISA (absorbance >1.0).

*^b^*Wells with antibodies that recognized free DBP (inhibition >50% by 5 µg/mL DBP). IcELISA was carried out with 1 µg/mL DBAP-BSA, and culture supernatants giving absorbances out of range were diluted until absorbance about 1.0.

*^c^*Hybridomas secreting antibodies with the lowest IC_50_ for DBP were stabilized and cloned.

### Optimization of icELISA

**Figure 4:**
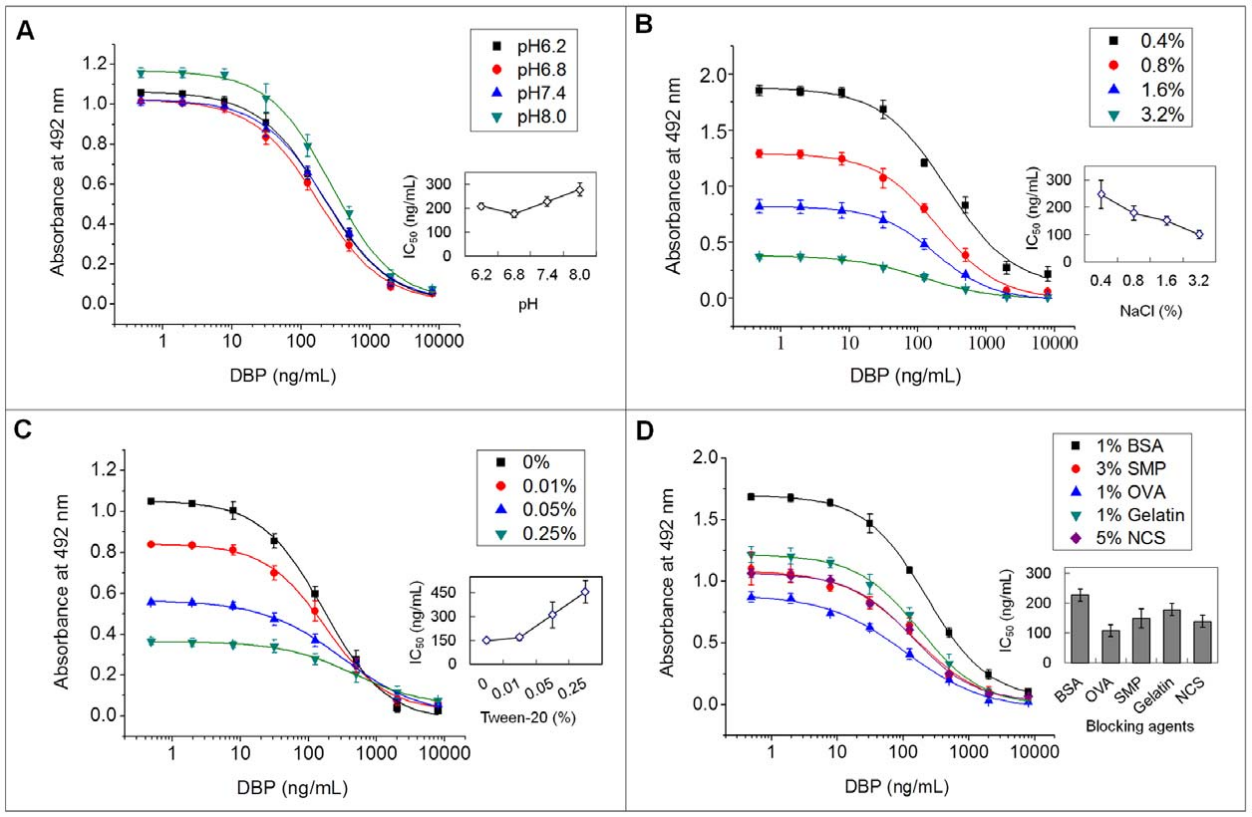
Effects of pH (A), ionic strength (B) and detergent (C) in assay buffer and blocking reagents (D) on icELISA for DBP. Each point represents the mean of ±SD of three replicates (three plates on the same day).

The use of organic solvents is very common in the ELISA of hydrophobic molecules to increase the solubility, so it is desirable to assess the effect of organic solvents on ELISA performance at the competition step. Different organic solvents such as N,N- dimethylformamide (DMF), dimethyl sulfoxide (DMSO), methanol, acetonitrile and dioxane were used to prepare DBP standard solution (1 mg/mL) and assay buffer (various amounts of organic solvents in PBS containing 0.1% BSA ). The effects of these organic solvents on the ELISA system are presented in Table 3. These solvents significantly influenced assay performance. It is interesting to note that although the maximum absorbance was enhanced by increasing the concentration of DMF, methanol and acetonitrile, an opposite trend was observed with DMSO. IC_50_ values in the presence of dioxane were much higher than those in the presence of other organic solvents. Fig. 4 presents the effect of pH, NaCl concentration, Tween-20 in assay buffer, and blocking agents on ELISA characteristics. Slightly acidic and neutral pH almost had no significant effect on A_max_, while slightly alkaline pH increased A_max_ and IC_50_ value. The lowest IC_50_ (177.04±15.37 ng/mL) was obtained at pH 6.8 (Fig. 4A), so this pH was selected for further optimization assays. The A_max_ was <0.5 when the NaCl concentration was 3.2%. An A_max_ of 0.8–1.0 was desirable, so the NaCl concentration in assay buffer selected as a compromise between signal and IC_50_ was 1.6% (Fig. 4B). Using the above optimized conditions, the effect of the detergent concentration was studied. As it is shown in Fig. 4C, lower A_max_ and higher IC_50_ values were observed as Tween-20 concentration increased, indicating that the addition of Tween-20 to the assay buffer did not enhance the sensitivity of the ELISA for the detection of DBP. Various blocking agents for each immunoassay need to be tested. As shown in Fig. 4D, OVA as a blocking reagent showed a higher sensitivity for the ELISA than other blockers, the IC_50_ value was 108.13±19.52 ng/mL. On the basis of these results, the optimal conditions for the icELISA were summarized as follows: OVA as a blocking reagent, the assay buffer used was 0.01 mol/L PBS containing 0.1% BSA, 10% DMF and 1.6% NaCl (pH 6.8). The most suitable combination of the immunoreagents was a coating antigen DBAP-BSA concentration at 0.25 µg/mL with dilutions of 1∶ 8000 (125 ng/mL) for the MAb tested by checkerboard titration.

**Table 3:** Influence of organic solvents in assay buffer on parameters of icELISA.

Organic Solvents	Concentration (%)	A_max_	IC_50_ (ng/mL)
DMF	0	0.501±0.0242	369±27.4
	5	0.831±0.0113	303±30.2
	10	1.056±0.0148	223±11.8
	20	0.922±0.0132	345±17.5
DMSO	0	0.849±0.0271	392±70.2
	5	0.563±0.0506	373±31.8
	10	0.614±0.0315	520±66.1
	20	0.538±0.0212	423±53.0
Methanol	0	0.647±0.0154	1400±64.3
	5	1.025±0.0127	637±67.1
	10	1.136±0.0349	624±14.5
	20	1.064±0.00930	889±18.6
Acetonitrile	0	0.493±0.0142	471±30.6
	5	0.972±0.0282	444±11.8
	10	1.068±0.0206	325±8.8
	20	1.097±0.0474	428±11.2
Dioxane	0	0.742±0.0312	797±19.8
	5	0.938±0.0419	1679±271
	10	0.684±0.0282	2509±504
	20	0.237±0.0253	2191±224

Each data represents the mean of ±SD of three replicates (three plates on the same day).

### Optimization of direct attachment of hapten in hapten coated ELISA

The amount of antibody on the modified polystyrene wells under different concentrations of EDC and conjugation temperatures was shown in Table 4. Treatment of oxidized polystyrene with 0.4% and 0.8% EDC gave almost the same results. The lower concentration (0.4% EDC) was chose for the standard procedures. The maximum binding of the antibody on the hapten-coated polystyrene surface occurred when hapten conjugation at 37°C overnight. EDC-mediated conjugation between carboxyl and amino usually carried out at 4°C or 25°C. However, the reaction between carboxyl and amino was not yet saturated after incubation at these temperatures in microwells. Thus 37°C was adopted as the conjugation temperature in the coating step. The optimal coating concentration of DBAP and dilution of MAb determined by checkerboard titration were 1 µg/mL and 1∶1000 (1 µg/mL), respectively.

**Table 4:** Absorbance of anti-DBP antibody bound to hapten-coated polystyrene wells.

	Conjugation temperatures		
EDC (%)	4°C	25°C	37°C
0.1	0.272±0.0213	0.573±0.0415	1.084±0.0351
0.2	0.386±0.0252	0.686±0.0253	1.478±0.0090
0.4	0.451±0.0416	1.011±0.0308	1.526±0.0283
0.8	0.462±0.0384	1.034±0.0552	1.520±0.0441

Absorbance at 492 nm under different concentrations of EDC and conjugation temperatures. Each data represents the mean of ±SD of three replicates (three plates on the same day).

### Standard Curves of icELISA for DBP

**Figure 5:**
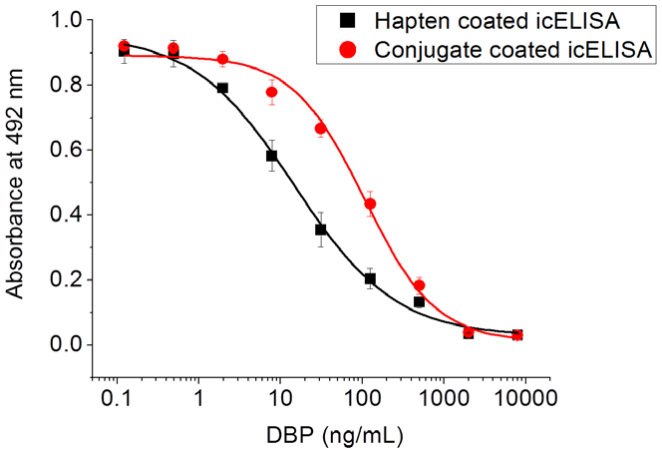
Representative icELISA standard curves for DBP. Each point represents the mean of ±SD of three replicates (one plate on three different days). The standard curve was calculated according to the 4-parameter equation *y* = (*A*−*D*)/[1+(*x*/*C*)*^B^*)]+*D* with the following values: for hapten coated icELISA, *A* = 0.956, *B* = 0.705, *C* = 14.6 ng/mL, *D* = 0.027, R^2^ = 0.9985; for conjugate coated icELISA, *A* = 0.892, *B* = 0.994, *C* = 106 ng/mL, *D* = 0.0094, R^2^ = 0.9976.

Fig. 5 exhibits typical standard curves of the hapten coated and conjugate coated icELISA for DBP under the above optimized conditions. In the conjugate coated icELISA, the IC_50_ value was 106 ng/mL, showing the LOD of 10.5 ng/mL and the dynamic range of 25.2–445 ng/mL. In the hapten coated icELISA, the IC_50_ value was 14.6 ng/mL with LOD of 0.426 ng/mL and dynamic range of 1.74–123 ng/mL. The sensitivity of icELISA for DBP in direct hapten coated format was significantly increased, which was about 7 folds higher than the assay performed with conjugate coated format.

### Monoclonal Antibody Specificity

The hapten was originally designed to generate specific antibody against DBP. To test this design we evaluated MAb recognition to some chemicals structurally resembling portions of DBP structure (Table 5). The most sensitive assay was obtained for DBP, and its CR was considered as 100%. For all of the other compounds tested, the CRs achieved were very low except that for BBP (21.3%) and the hapten DBAP (27.8%).

**Table 5:** Cross-reactivity of some compounds structurally related to DBP by hapten coated icELISA.

Analyte	IC_50_ (ng/mL)^a^	CR (%)
DBP	14.7±2.8	100
DBAP	52.9±12.4	27.8
BBP	72.1±14.2	21.3
DMP	-	7.98% inhibition at 10 mg/mL
DEP	-	5.08% inhibition at 10 mg/mL
DEHP	-	1.12% inhibition at 10 mg/mL
DOP	-	1.15% inhibition at 10 mg/mL

*^a^*Each data represents the mean of ±SD of three replicates (three plates on the same day).

### Recovery of DBP Spiked into Water Samples

Water samples from two different sources were spiked with DBP covering the optimized working range (10, 50, and 100 ng/mL) and analyzed by the hapten coated icELISA with the MAb. Determinations were made in triplicate. Recoveries from tap water and East Lake water are presented in Table 6. Without spike, all water samples were analyzed as negative (below LOD). The recoveries of DBP from spiked tap water and East Lake water were in ranges of 96.8–117% and 95.2–105%, respectively. In respect of reproducibility, the intra-assay coefficients of variation (CV) values varied from 6.20% to 11.8%.

**Table 6:** Recovery of DBP spiked into water samples.

Samples	Spiking (ng/mL)	Dectected (ng/mL)^a^	Recovery (%)	Coefficient of variation (%)
Tap water	0	<LOD	-	-
	10	11.7±1.38	117	11.8
	50	48.4±4.89	96.8	10.1
	100	111±11.8	111	10.5
East Lake Water	0	<LOD	-	-
	10	10.5±1.14	105	10.8
	50	47.6±2.94	95.2	6.20
	100	101±10.34	101	10.3

*^a^*Each data represents the mean of ±SD of three replicates (the same sample run on one plate on three different days).

### Content of DBP in some consumer products

**Figure 6:**
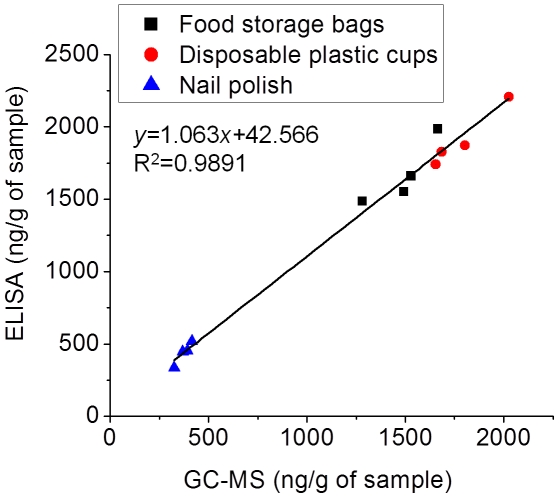
Correlation of ELISA and GC-MS analysis of certain household items. Each point represents the mean of three replicates of one sample.

The hapten coated icELISA was applied to determine DBP in food storage bags, disposable plastic cups and nail polish samples from a supermarket in Wuhan, China. Good agreement (The linear equation was *y* = 1.063*x*+42.566, R^2^ = 0.9891) was observed between the results obtained by the hapten coated icELISA and GC-MS (Fig. 6), further confirming the reliability of the hapten coated icELISA for DBP.

## Discussion

In theory, an introduction of spacer with a certain length is conducive to the generation of antibodies with high titer and sensitivity, especially for hydrophobic small molecules [28]. In this study, two DBP derivatives DBAP and DBCP, which differed in the spacer length, were used for conjugation with carrier proteins. Antibodies raised against DBCP-protein conjugates showed higher titer but much lower sensitivity than DBAP-protein conjugates, which implied that haptens with no or shorter spacer length gave better sensitivity than the longer ones. Brun et al and Mercader et al had also reached the same conclusion [29], [30]. As BSA possesses higher molecular weight, more lysine residues, more stable physical and chemical properties, and better solubility than OVA, it is the preferred carrier for the synthesis of artificial antigen [31]. However, DBAP-OVA promoted antibodies with higher sensitivity than DBAP-BSA, which suggested that OVA might be a better carrier than BSA for immunogen synthesis of DBP.

MAb production with lymphocyte hybridoma technique is mature nowadays and advantages of MAb are obvious. However, polyclonal antibody but not MAb was used in most immunoassays for small organic molecules. Manclús et al [32] found that highly sensitive rabbit polyclonal antibody to chlorpyrifos had been obtained using a derivative of chlorpyrifos-methyl to prepare the immunogen, but attempts to produce MAb with the appropriate properties from the same hapten were unsuccessful. This phenomenon may due to the ineffective antibody raised from mice with the same immunization protocol. To avoid this case, a simply modified immunization procedure described by Hu et al [14] was adopted in this study. With two continual injections of the emulsion of antigen and Freund's complete adjuvant within three days, it resulted in satisfactory titer and sensitivity of the antisera from mice within two months.

Various factors may interfere with the antigen-antibody interaction, causing differences between expected and observed ELISA results. Accordingly, we studied organic solvent, pH, ionic strength, detergent, and blocking reagent as possible sources of interference in the ELISA with DBP MAb. Most researchers reached the same conclusion that methanol caused the least negative effect on the ELISA performance [33]–[35]. However, our assays showed that DMF presented the best combination of A_max_ and IC_50_. So DMF was selected for the cosolvent in the ELISA for DBP. The ELISA for DBP was very sensitive to ionic strength, that is, A_max_ and IC_50_ values significantly decreased at high concentration of NaCl. The sensitivity improvement by increased ionic strength may be due to dispersion and weakening of the nonspecific binding derived from antibody [35]. Tween-20 is a nonionic detergent commonly used in washing buffer to reduce nonspecific interactions in ELISA. However, in this study, lower A_max_ and higher IC_50_ values were observed as the concentration of Tween-20 in assay buffer increased. This fact may be related to nonspecific hydrophobic interactions between the detergent and nonpolor small analytes in an aqueous environment, thereby interfering with the specific analyte-antibody interaction [36]. Thus Tween-20 was not used in the ELISA for DBP. Skim milk as a blocking agent often shows a lower background signal and a higher sensitivity for ELISA than other blockers [37], [38]. Although OVA as a blocking agent slightly reduced detection signal, the sensitivity for the ELISA for DBP was increased obviously. It was demonstrated again that there is no universal blocking agent for all immunoassays.

Although methods had already been reported for linking hapten to polystyrene surface [9], [11], it was not applicable to the hapten coated ELISA for DBP because the MAb produced hardly bound to the carboxylated hapten DBCP on polystyrene (The absorbance value at 492 nm was too low.). Thus it was needed to immobilize the aminated hapten DBAP to the surface of polystyrene. Holthues et al had linked an aminated hapten ABA–atrazine to a glutaraldehyde polymer network directly bound to polystyrene surface and developed very sensitive ELISA to detect terbutryn [10]. However, it was a failure to establish hapten coated ELISA for DBP based on covalently linking DBAP to a glutaraldehyde polymer network on polystyrene surface. We found that DBAP poorly reacted with glutaraldehyde polymer network in 96-well microtiter plate. Therefore a novel method of linking aminated hapten to carboxyl group on polystyrene surface with EDC was developed, which was demonstrated to be successful in the hapten coated icELISA for DBP. The hapten coated format presented is proposed as generally applicable, because the carboxyl on modified microtiter plate surface enables stable immobilization of aminated or hydroxylated hapten through EDC. Meanwhile, the IC_50_ in direct hapten coated format was about 7 folds lower compared with conjugate coated format, which was significantly increased the sensitivity of icELISA for DBP.

In order to investigate the specificity of the MAb produced, CR was determined between DBP and its structural analogues. For all of the compounds tested, the CRs achieved were very low except that for BBP and the hapten DBAP. The recognition pattern of the competitors showed the great importance of the simultaneous presence of a phthalyl group and the butyl ester group in the chemical structure of the analyte. Thus, the absence of recognition of several phthalates, such as DMP, DEP, DEHP and DOP, demonstrated that the presence of a phthalyl group alone was not sufficient for antibody recognition. Generally, the sensitivity and CR of antibody are dependent on several factors such as the structure of immunogen and the physicochemical properties of the test analytes. Electrostatic and hydrophobic interaction can determine the strength of binding and CR through the difference of electron distribution between haptens [35]. MAb was raised against DBAP-OVA conjugate in this study. In theory, CR caused by DBAP should be higher than DBP. However, it was interesting to find that DBP combined with the MAb more easily than DBAP and the CR caused by DBAP was only 27.8% of DBP. It may be due to the reason that the amido of DBAP is electron negative which increases the electron density on the benzene ring. Nevertheless, when DBAP coujugated with protein via diazotization, the electron density on the benzene ring was transfer dispersion because of aromatic resonance with nitrogen-nitrogen double bond (N = N). Therefore, the MAb was easier to recognize DBP which had relatively low electron density on the benzene ring compared with DBAP.

Spiking matrix samples with several amounts of analyte is a common practice to perform a preliminary evaluation of analytical assay reliability. Hence, water samples from two different sources were spiked at several concentrations of DBP covering the established working range. The mean recovery for two environmental waters at three levels of fortification was 104%. Concerning the reproducibility, the average intra-assay CV was 9.95%. The results demonstrate that the developed hapten coated icELISA is suitable for the detection of DBP in tap water and fresh water.

Compared with GC-MS, ELISA still has drawbacks that may give an overestimated value because of the CR. The contents of DBP in three household items detected by ELISA are all higher than GC-MS, which may due to the CR caused by BBP. However, ELISA offers considerable advantages over the conventional analytical procedure because of its efforts for sample cleanup, relatively fast measurement, high throughput detection, and acceptable costs. With these important and attractive features, ELISA developed in this study can contribute to the simultaneously routine monitoring of DBP in water, plastic and cosmetic samples. In the past, certain research institutions believed that DBP was not used as a plasticizer in PVC plastics [39]. However, our research found that DBP was really existed in some PVC products such as food storage bags and disposable plastic cups and the contents of them were nearly up to 2000 ng/g, which indicated that DBP was used extensively for PVC plastics.
